# The functional switch of TGF-β signaling in breast cancer

**DOI:** 10.18632/oncotarget.26715

**Published:** 2019-02-26

**Authors:** Qiaoni Shi, Ye-Guang Chen

**Affiliations:** Ye-Guang Chen: The State Key Laboratory of Membrane Biology, Tsinghua-Peking Center for Life Sciences, School of Life Sciences, Tsinghua University, Beijing, China

**Keywords:** TGF-β, Smad3, HER2, EGFR, functional switch

TGF-β (transforming growth factor β) has a dichotomous role in breast cancer development: it suppresses breast cancer initiation by blocking cell proliferation at earlier stages, while enhancing cancer progression and malignancy through promoting epithelial-mesenchymal transition (EMT) and cell migration in advanced stages [1]. However, the mechanistic basis of TGF-β function switch in cancer was elusive. HER2/EGFR (epithelial growth factor receptor) signaling promotes cell survival and proliferation, and overexpression or hyperactivation of EGFR family members exacerbates breast cancer malignancy [2, 3]. It has long been noted that TGF-β signaling and HER2/EGFR signaling collaborate and modulate each other at various levels during breast cancer development [3, 4]. TGF-β induces survival, migration and invasion of HER2-overexpressed epithelial cells and increases drug resistance; TGF-β also regulates the expression of ligands and receptors in the HER2/EGFR pathway. Conversely, HER2/EGFR signaling controls TGF-β ligand expression and Smad-dependent transcription.

Unlike in other cancers such as colorectal and pancreatic carcinoma, core components of the TGF-β pathway such as TβRII and Smad4 are rarely mutated in breast cancer, but the tumor-suppressive action of TGF-β is still preferentially disabled. HER2/EGFR signaling has been shown to impair the cytostatic function of TGF-β. For instance, the transcription factor C/EBPβ is important to mediate the TGF-β-induced cytostasis by controlling the expression of p15 and c-myc, and HER2 overexpression enhances the expression of the dominant-negative form of C/EBPβ and leads to TGF-β resistance in breast cancer cells [5].

**Figure 1:**
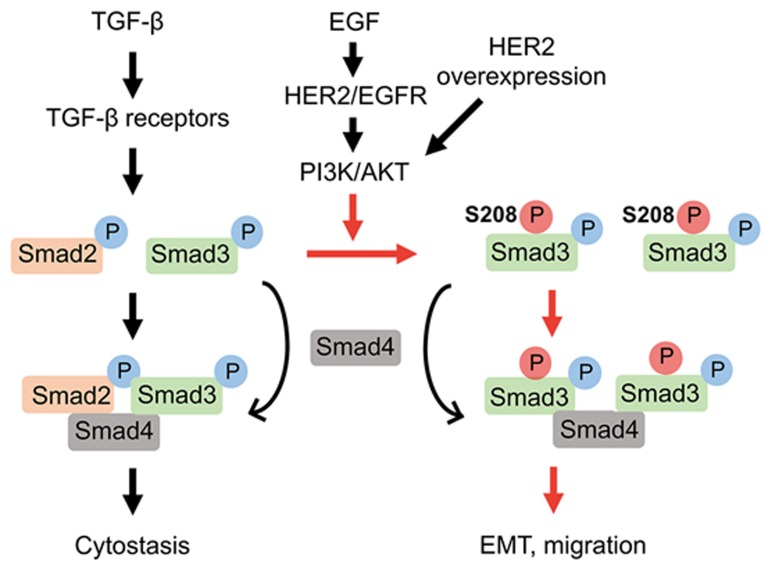
HER2/EGFR signaling silences the tumor-suppressive function of TGF-β while promoting its effect on EMT and cell migration HER2/EGFR activation or overexpression promotes the linker phosphorylation at S208 of Smad3 by AKT, enhances Smad3 retention in the nucleus and upregulates the genes related to EMT, migration to switch TGF-β function from anti-proliferation to breast cancer promotion.

Although the interplay between TGF-β signaling and HER2/EGFR signaling has been well appreciated, it was unclear whether HER2/EGFR signaling could directly modulate TGF-β signaling and thus switch TGF-β function. In our study [6], we discovered that HER2/EGFR turns TGF-β function from anti-proliferation to the pro-invasion by controlling the nuclear localization of Smad3 (Figure 1). High HER2/EGFR activity increases AKT-mediated S208 phosphorylation in the Smad3 linker region, which prolongs the nuclear retention of Smad3. The regulation of HER2/EGFR-AKT signaling on TGF-β is Smad3-specific as it has no effect on Smad2. High Smad3 activity or a high Smad3/Smad2 ratio enhances the expression of EMT- and migration-related genes. This regulatory mechanism takes place in both HER2-high or triple negative breast cancer cells that express HER2 at the physiological level.

Our study elicits several unsolved issues that need further investigation. Firstly, as the crucial cytoplasmic signal transducers of TGF-β signaling, Smad2 and Smad3 have been indicated to exert different physiologic functions in different circumstances [1, 6–8]. Smad2 seems to have essential functions to mediate Activin/Nodal signaling during embryogenesis, while Smad3 mediates TGF-β responses in adult tissues, such as growth inhibition, fibrotic responses in response to injury, synthesis of extracellular matrix proteins, immune suppression, EMT and cell migration [1, 7, 8]. However, as most cells in adult tissues express both Smad2 and Smad3, the importance of the ratio of Smad2 *vs*. Smad3 in TGF-β-mediated responses is unclear, and needs further exploration. Secondly, there are multiple phosphorylation sites in the linker region of Smad3, and various kinases have been shown to phosphorylate these sites [8]. The upstream signals to regulate linker phosphorylation and the functional outputs are complex and warrant future investigation. Lastly, due to the involvement of TGF-β signaling in human diseases such as tissue fibrosis and carcinogenesis, great attempts have been making to develop drugs to modulate this pathway, including anti-ligand antisense oligonucleotides, ligand competitive peptides, antibodies that target ligands, receptors or associated proteins, and small molecular inhibitors against TGF-β receptor kinase [9]. However, these approaches universally block all effects of TGF-β and cause unwanted side effects. Development of Smad3-specific small molecular inhibitors or selective Smad3-interacting peptide aptamers would be a reasonable approach.
